# Effect of the Purple Corn Beverage “Chicha Morada” in Composite Resin during Dental Bleaching

**DOI:** 10.1155/2016/2970548

**Published:** 2016-03-01

**Authors:** Eric Dario Acuña, Leyla Delgado-Cotrina, Francisco Aurelio Rumiche, Lidia Yileng Tay

**Affiliations:** ^1^Odontología Restauradora y Estética, Facultad de Estomatologia, Universidad Peruana Cayetano Heredia, Lima, Peru; ^2^Ingeniería de Materiales, Facultad de Ingenieria Mecánica, Pontificia Universidad Católica del Perú, Lima, Peru

## Abstract

During dental bleaching the staining potential of the surface would increase. This study aims to evaluate the staining susceptibility of one bleached composite resin after the exposure to three different beverages: Peruvian purple corn based beverage (chicha morada), green tea, and distilled water. Thirty disk-shaped specimens of one nanofill composite resin were prepared. The specimens were then divided into six groups (*n* = 5): purple corn (P), purple corn + bleaching (PB), green tea (T), green tea + bleaching (TB), distilled water (W), and distilled water + bleaching (WB). In groups that received bleaching, two sessions of bleaching with 35% hydrogen peroxide were done. Following bleaching, specimens were exposed to each liquid thirty minutes daily. Color was measured with a digital spectrophotometer. For statistical analysis, color measurement differences between the obtained results were used: during bleaching, after bleaching, and during + after bleaching. Two-way ANOVA was used to compare the color changes in the resins of all groups (*p* < 0.05). We conclude that all the evaluated beverages produced changes of color in the composite resin regardless of the bleaching procedure. However, purple corn was the only beverage that caused a perceptible color change (Δ*E* > 3.3).

## 1. Introduction

Different authors have studied the influence of beverages in the restorative materials color [1, 2]. Several dark beverages have been studied, including red wine [3–5], black tea [6–9], coffee [1, 6, 8–10], carbonated drinks [1, 8, 9], and natural juices [4, 5, 11]. Bleaching agents, such as hydrogen and carbamide peroxide, can successfully remove the stains generated by dark beverages in composite resins [3, 7, 8, 10, 12]. However, researches had also showed that bleaching could affect the roughness [10, 13, 14] and hardness [15–18] of the surface of dental resin composite. It would be expected that during dental bleaching the staining potential of the surface would increase; on the other hand some studies have shown that the exposure to dark beverages during bleaching does not affect the bleaching result [3, 7, 8, 10, 12].

In Peru several beverages that are made from native products are consumed daily. One of them is chicha morada, a dark nonalcoholic beverage originated in the Andes [19]. Chicha morada is one of the most consumed beverages in the country [19]. It is usually made by boiling Culli/Ckolli type purple corn (*Zea mays indurata*) [20] with different ingredients: pineapple, cinnamon, clove, lemon, and sugar. However, there are no studies about the effect of purple corn in the color of resin composites.

The present study aims to evaluate the staining susceptibility of a composite resin during and after bleaching with 35% hydrogen peroxide immersed daily in a purple corn based beverage.

## 2. Materials and Methods

### 2.1. Specimen Preparation

Thirty disk-shaped specimens were made of a nanofill resin composite (Filtek Z350 XT, 3M ESPE, Saint Paul, EEUU), A2 body shade. Specimens were built up in one increment (2 mm) into a metal mold (7 mm in diameter and 2 mm in thickness). The mold and the material were then covered with Mylar strips on top and bottom and placed between two glass slides. Finger pressure was then applied to extrude excess material. The composite resin was then light-cured with a LED unit (Valo*®*, Ultradent, South Jordan, EEUU) for 20 s with a light intensity of 1000 mW/cm^2^. Intensity was checked with a radiometer (Demetron LED Radiometer, Kerr, Orange, EEUU). Distance between the LED unit and the specimen was standardized by 1 mm glass slide. Then all specimens were stored in distilled water at room temperature.

Specimens were divided into six groups (*n* = 5): purple corn without bleaching (P), purple corn with bleaching (PB), green tea without bleaching (T), green tea with bleaching (TB), distilled water without bleaching (W), and distilled water with bleaching (WB) (Table 1).

### 2.2. Staining Substances

Standardized proportions were used for preparing the staining substances. For the purple corn based beverage, two purple corncobs were boiled for 10 minutes in 500 mL of distilled water. And for green tea, one tea bag was boiled for five minutes in 250 mL of distilled water (a cup) [8]. In both cases the pigmenting substances were allowed to cool until room temperature.

### 2.3. Staining Procedure

Specimens were daily immersed in 20 mL of the staining solutions (purple corn based beverage, green tea, or distilled water) for 30 minutes at room temperature in a container; the staining procedure was performed during the whole study during 35 days. After the staining procedure, the specimens were washed with distilled water and then stored in labeled containers with distilled water and held at room temperature during the study.

### 2.4. Bleaching Procedure

Bleaching was performed applying a 35% hydrogen peroxide bleaching agent (Whiteness HP Maxx, FGM, Joinville, Brazil) on the sample surface. Two sessions were performed, with one-week interval between sessions. In each session the bleaching agent was applied twice for 15 minutes each. After each application the gel bleaching agent was removed, washed with distilled water, and stored in distilled water until other procedures.

### 2.5. Color Measurement

Color specimens were measured with a digital spectrophotometer (VITA Easyshade Advance 4.0, VITA Zahnfabrik, Bad Säckingen, Germany). Following the manufacturer instructions, a calibration with a B1 color block was performed before beginning the readings. The measurements were performed at the same hour of the day in the same room against a white background [7]; a silicon mold was made to standardize the angle of measurement. The specimens were dried with absorbent paper before the reading.

The color difference (Δ*E*) values were recorded as given by the digital spectrophotometer. The *L* ^*∗*^ (lightness), *a* ^*∗*^ (−*a* ^*∗*^ = green; +*a* ^*∗*^ = red), and *b* ^*∗*^ (−*b* ^*∗*^ = blue; +*b* ^*∗*^ = yellow) values were also recorded.

Three measurements were performed for each specimen in seven different times: T0—control (before the first session of bleaching), T1—after the first session of bleaching, T2—before the second session of bleaching, T3—after the second session of bleaching, T4—1 week, T5—2 weeks, and T6—4 weeks.

### 2.6. Statistical Analysis

For statistical analysis, color measurement differences among the obtained results were used: T3–T0 (during bleaching), T6–T3 (after bleaching), and T6–T0 (during + after bleaching). The differences were analyzed using the statistical software BioStat v5. Two-way ANOVA was used to compare the color changes in the resins of all groups (*p* < 0.05). Multiple comparisons of data were analyzed with Tukey post hoc test.

## 3. Results

**Figure 1:**
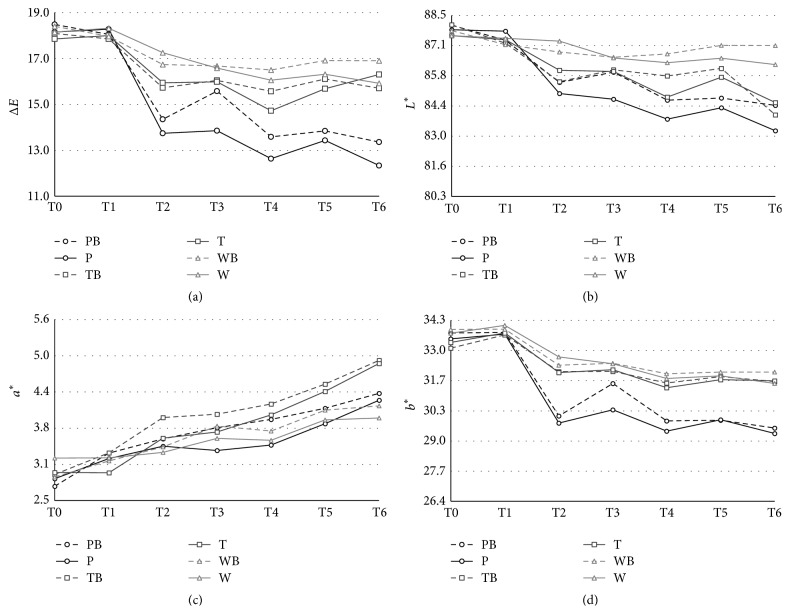
(a) Δ*E* values after staining and bleaching with 35% hydrogen peroxide. (b) *L* ^*∗*^ values after staining and bleaching with 35% hydrogen peroxide. (c) *a* ^*∗*^ values after staining and bleaching with 35% hydrogen peroxide. (d) *b* ^*∗*^ values after staining and bleaching with 35% hydrogen peroxide. PB: purple corn + bleaching; P: purple corn; TP: green tea + bleaching; T: green tea; WB: distilled water + bleaching; W: distilled water; T0: control; T1: after the first session of bleaching; T2: before the second session of bleaching; T3: after the second session of bleaching; T4: 1 week; T5: 2 weeks; and T6: 4 weeks.

In this study all evaluated beverages decreased the Δ*E* of dental composite resins (Figure 1(a)). The exposure to purple corn caused major statistically significant color differences when compared with green tea and distilled water regardless of the bleaching agent exposure (*p* < 0.05). During the first week of exposure to different beverages the purple corn beverage caused the largest decrease of Δ*E*, followed by PB (*p* < 0.05); however, after a month of exposure there were no significant differences in the decrease of Δ*E* in both groups. There were no significant differences in the decrease of Δ*E* between groups exposed to green tea and distilled water regardless of the bleaching agent exposure (Table 2).

Regarding the lightness results (*L* ^*∗*^), all substances evaluated decreased *L* ^*∗*^ of the evaluated composite resin (Figure 1(b)). Purple corn and green tea generated statistically significant differences compared to distilled water (*p* < 0.05). During the first week of exposure (bleaching exposure) the exposure to purple corn generated the major decrease of lightness (*p* < 0.05); however, after one month of exposure to the substances there were no significant differences in the decrease of *L* ^*∗*^ between groups exposed to purple corn and green tea, regardless of the use of the bleaching agent. There were no significant differences in the decrease of *L* ^*∗*^ in the control group with or without bleaching (Table 2).

When evaluating *a* ^*∗*^ (negative values: green; positive values: red), it was found that all evaluated substances increased *a* ^*∗*^ of composite resins (Figure 1(c)). Green tea exposure caused a greater increase in *a* ^*∗*^. However, there are no statistically significant differences compared to distilled water or purple corn. During the bleaching there was a greater increase in *a* ^*∗*^ in groups exposed to bleaching agent; however, after one month of exposure to the beverages there were no significant differences in the increase of *a* ^*∗*^ among any groups. The increase of *a* ^*∗*^ was lower when exposed to distilled water (Table 2).

Finally, *b* ^*∗*^ values were evaluated (negative values: blue; positive values: yellow), all evaluated substances decreased *b* ^*∗*^ of the evaluated composite resin (Figure 1(d)). Purple corn beverage caused statistically significant differences compared to green tea and distilled water (*p* < 0.05). During the bleaching the group exposed to purple corn generated the higher decrease, followed by the PB group (*p* < 0.05); however, after a month of exposure to the beverages there were no significant differences between both groups. There were no significant differences between the decrease of *b* ^*∗*^ and the groups exposed to green tea and distilled water, regardless of whether it was exposed to the bleaching agent or not (Table 2).

## 4. Discussion

The results demonstrated that all the tested beverages produced changes in color of the evaluated composite resin, regardless of the bleaching procedure.

Composites are susceptible to staining when exposed to different beverages [11]. It has been found that the beverages with the highest potential of staining are red wine [3–5], black tea [6–9], and coffee [1, 6, 8–10]. There are also reports of color change produced by the exposure to carbonated drinks [1, 8, 9] and juices such as guaraná [8], grape [8], and açaí [11].

The composition of dental composites resins can promote staining. Composite resins have dimethacrylate monomers such as bisphenol A (Bis-EMA) [11] and triethyleneglycol dimethacrylate (TEGDMA) [5, 21]; studies show that when the ratio of these components is increased the composite resins become stained more quickly. Both monomers are found in Filtek Z350 XT, which is the composite resin used in this study. Furthermore, it has been shown that nanofill resins, such as Filtek Z350 XT, have more staining potential compared to microhybrid and nanohybrid resins [5]; this may be explained because their particles are easily lost due to their size, creating spaces, which are deposit sites for pigments. The filler particles are detached as a result of the hydrolysis, generating an increase of opacity and change of appearance of the dental composite resin [22].

This study aims to evaluate the in vitro effect of the exposure to a purple corn beverage on the color of a bleached dental composite resin. This study only assessed the pigmenting effect of the Andean purple corn, the main ingredient of the popular drink “chicha morada.” This beverage is prepared by mixing the purple corn with other ingredients, such as lemon and sugar [19], that could affect the results of the present study. Further studies should analyze the effect of these beverages in the dental composite resin.

The effect of staining with “chicha morada” (or purple corn) on dental composites has not been studied. In this study we found that the purple corn beverage was the drink that generated the higher color changes (Δ*E*). These results are explained because purple corn has a pigment called anthocyanin [20]; depending on the pH of the beverage it turns red, purple, or blue [11]. Other beverages that contain anthocyanin are blueberries, red grapes, red wine, and açaí [5]. According to the literature one of the drinks that cause the greatest pigmentation is red wine (fermented drink made from grape juice). This increased pigmentation may be explained due to the presence of alcohol [5] and low pH [3]; this could degrade the surface becoming more susceptible to stains [3]. Açaí is a fruit from Brazil consumed in juices, just as “chicha morada.” Different authors have found that the composites had lower pigmentation when exposed to açaí, when compared with coffee [11] and red wine [5].

During this study we used the VITA Easyshade Advance 4.0 digital spectrophotometer (VITA, Germany). The use of spectrophotometers reduces failures in the visual measurement method [23] because this may be affected by age, sex, skill, and psychological conditions of the individual who performs it [24]. Studies had found that the use of spectrophotometers improves by 33% the accuracy and helps obtaining objective results in 93.3% of cases [25]. We should note that the color of the composite resin samples used in this study was A2 according to the manufacturer but when initial measures were registered by the digital spectrophotometer a B3 color was obtained. Although different manufacturers use the same nomenclature, it is common to find different shades in resins.

It is important to evaluate the changes in Δ*E*; however it is also important to analyze the changes in the different dimensions of color because this will help us understand what is really changing in the surface of the composite [26]. In this study, *L* ^*∗*^ decreased after exposure to purple corn beverage, implying that composite resins became darker. Also we found a greater *b* ^*∗*^ decrease; this means that resins became bluer.

In this study, composite resins exposed to purple corn beverage had a difference in Δ*E* of more than 5. According to Ruyter et al. 1987, this is a human eye perceptible change, since Δ*E* changes under 3.3 are not perceptible to the human eye [27]. However, exposure to purple corn during bleaching (T3–T0) had no detectable changes, compared to the group exposed to purple corn without bleaching during the same period of evaluation. This is similar to other studies that show that staining beverages do not affect the composite staining susceptibility during dental bleaching [3, 7, 8, 10]. It is worth mentioning that similar values were found in both groups when evaluating the total difference after exposure to purple corn for a month and a half.

Regarding the green tea, no statistically significant differences in color (Δ*E*) compared with distilled water were found; in both cases changes were not clinically detectable (Δ*E* less than 3.3). When evaluating the different color dimensions we found that *L* ^*∗*^ (brightness) and *b* ^*∗*^ (turned slightly blue) decreased, although *b* ^*∗*^ presents lower changes compared to exposure to purple corn. A greater statistically significant variation of *a* ^*∗*^ was observed; this means the composite became more red.

Black tea is considered one of the beverages with higher pigmentation effect on composite resins [6–9]. However, there are not many articles about the effect of green tea on the color of a composite resin. Green tea consumption has increased over the years because of its higher level of antioxidants [28] and it can be also consumed at room temperature and cold, like “chicha morada.” Tan et al., 2015, also compared the effects of different beverages, concluding that coffee generally produced the worst stains, followed by red wine and green tea. According to Tan et al., the change of Δ*E* generated by green tea was not perceptible and *L* ^*∗*^ and *a* ^*∗*^ decreased and *b* ^*∗*^ increased [1].

In the control group there were changes in Δ*E* of the evaluated composite resin, even when it was not exposed to any pigmenting agents. These changes were not clinically detectable; this can be explained due to more water sorption in the first 7 days [29], and this explains why the reduction of Δ*E* stabilizes. When we evaluate the effect of dental bleaching in different dimensions of color, we find higher *a* ^*∗*^ values in all the bleaching groups; this means the composite becomes more red. However, all of these changes were not perceptible.

Stains can be observed clinically in dental tissue and restorative materials. Bleaching with hydrogen or carbamide peroxide [30] has been demonstrated to be effective for removing staining of red wine [3], black tea [7, 8], and coffee [3, 8, 10] from composite resin restorations.

Few studies evaluate the effect of bleaching agents in composite resins, each having different methodology [8]. The diverse methodologies used to evaluate color make it difficult to do comparisons; therefore further studies should be conducted with standardized methodology. Four of the reviewed studies evaluated the effect of bleaching in composite resins by pigmenting the resins with different beverages (coffee [3, 8, 10], black tea [7, 8], red wine [3], or a soft drink [8]) and then evaluate the effect of different bleaching agents. Çelik et al. evaluated the susceptibility to pigmentation after bleaching by applying 20% carbamide peroxide first for 8 days and then pigmenting the composite with black tea or coffee for 30 days, finding that both substances had the same pigmentation susceptibility regardless of whether the composite underwent dental bleaching or not [6]; although scientific evidence reports changes in microhardness and roughness of the composite resin surface after bleaching, that may make us expect higher pigmentation in bleached composite resin. These results are similar to the findings in this study, where a similar methodology was used with in-office agent (35% hydrogen peroxide).

## 5. Conclusions

Within the limitations of this in vitro study, all beverages (purple corn, green tea, and distilled water) generated color changes in the evaluated composite resin. Exposure to purple corn beverage was the only beverage that generated pigmentations noticeable to the human eye in restorations based on composite resin. Exposure to purple corn beverage during bleaching procedure with 35% hydrogen peroxide generates color changes, but this change is not perceptible to the human eye.

## Figures and Tables

**Table 1:** Groups.

Group	
P	Samples exposed to purple corn
PB	Bleached samples exposed to purple corn
T	Samples exposed to green tea
TB	Bleached samples exposed to green tea
W	Samples exposed to distilled water
WB	Bleached samples exposed to distilled water

**Table 2:** Media of Δ*E*, *L* ^*∗*^, *a* ^*∗*^, and *b* ^*∗*^ at different times among groups.

		PB	P	TB	T	WB	W
Δ*E*	T3–T0	−2.9^A^	−4.3^B*∗*^	−2.0^C^	−1.9^C^	−1.7^C^	−1.6^C^
	T6–T3	−2.2^A^	−1.5^AB^	−0.3^BC^	0.3^C^	0.2^C^	−0.7^AC^
	T6–T0	−5.1^A*∗*^	−5.8^A*∗*^	−2.4^B^	−1.5^B^	−1.5^B^	−2.2^B^

*L* ^*∗*^	T3–T0	−2.1^A^	−3.2^B^	−2.1^AC^	−1.6^ACD^	−1.2^D^	−1.0^D^
	T6–T3	−1.5^A^	−1.4^AB^	−2.1^ABC^	−1.4^ABC^	0.5^D^	−0.3^D^
	T6–T0	−3.7^A^	−4.6^AB^	−4.1^ABC^	−3.0^AC^	−0.7^D^	−1.3^D^

*a* ^*∗*^	T3–T0	1.0^A^	0.5^B^	1.0^AC^	0.7^D^	0.9^ACD^	0.3^BE^
	T6–T3	0.6^A^	0.9^AB^	0.9^ABC^	1.2^BC^	0.3^AD^	0.3^AD^
	T6–T0	1.6^A^	1.4^AB^	2.0^AC^	1.9^AC^	1.2^AB^	0.7^D^

*b* ^*∗*^	T3–T0	−2.2^A^	−3.1^B^	−1.0^C^	−1.2^C^	−1.5^C^	−1.3^C^
	T6–T3	−2.0^A^	−1.0^B^	−0.4^C^	−0.5^C^	−0.4^C^	−0.9^C^
	T6–T0	−4.2^A^	−4.2^A^	−1.5^B^	−1.7^B^	−1.9^B^	−2.2^B^

Data followed by different letters at the horizontal are statistically significantly different by Tukey test (*p* < 0.05). *∗* indicates clinically unacceptable value (Δ*E* > 3.3). PB: purple corn + bleaching; P: purple corn; TB: green tea + bleaching; T: green tea; WB: distilled water + bleaching; W: distilled water; T3–T0: during bleaching; T6–T3: after bleaching; and T6–T0: during + after bleaching.

## Clinical Relevance

Patients with composite restorations should avoid “chicha morada” because of its higher staining susceptibility.

## References

[1] Tan B. L., Yap A. U., Ma H. N., Chew J., Tan W. J. (2015). Effect of beverages on color and translucency of new tooth-colored restoratives. *Operative Dentistry*.

[2] Omata Y., Uno S., Nakaoki Y. (2006). Staining of hybrid composites with coffee, oolong tea, or red wine. *Dental Materials Journal*.

[3] Villalta P., Lu H., Okte Z., García-Godoy F., Powers J. M. (2006). Effects of staining and bleaching on color change of dental composite resins. *Journal of Prosthetic Dentistry*.

[4] Tonetto M. R., Santezi C., Magnani C., Aleixo dos Santos P., Alves E., Marcelo F. (2012). Effect of staining agents on color change of composites. *RSBO Revista Sul-Brasileira de Odontologia*.

[5] De Alencar M. L., Da Cunha F. D., Meireles S. S., Duarte R. M., Andrade A. K. (2014). The effect of drinks on color stability and surface roughness of nanocomposites. *European Journal of Dentistry*.

[6] Çelik Ç., YÜzÜgÜllÜ B., Erkut S., Yazici A. R. (2009). Effect of bleaching on staining susceptibility of resin composite restorative materials. *Journal of Esthetic and Restorative Dentistry*.

[7] Poggio C., Beltrami R., Scribante A., Colombo M., Chiesa M. (2012). Surface discoloration of composite resins: effects of staining and bleaching. *Dental Research Journal*.

[8] Garoushi S., Lassila L., Hatem M. (2013). Influence of staining solutions and whitening procedures on discoloration of hybrid composite resins. *Acta Odontologica Scandinavica*.

[9] Nasim I., Neelakantan P., Sujeer R., Subbarao C. V. (2010). Color stability of microfilled, microhybrid and nanocomposite resins—an in vitro study. *Journal of Dentistry*.

[10] Mendes A. P. K. F., de Oliveira Barceleiro M., dos Reis R. S. A., Bonato L. L., Dias K. R. H. C. (2012). Changes in surface roughness and color stability of two composites caused by different bleaching agents. *Brazilian Dental Journal*.

[11] Costa e Silva D. D., Tiradentes S. B. D. S. P., Parente R. C. P., Bandeira M. F. C. L. (2009). Color change using HSB color system of dental resin composites immersed in different common Amazon region beverages. *Acta Amazonica*.

[12] Hafez R., Ahmed D., Yousry M., El-Badrawy W., El-Mowafy O. (2010). Effect of in-office bleaching on color and surface roughness of composite restoratives. *European Journal of Dentistry*.

[13] Wang L., Francisconi L. F., Atta M. T. (2011). Effect of bleaching gels on surface roughness of nanofilled composite resins. *European Journal of Dentistry*.

[14] Polydorou O., Hellwig E., Auschill T. M. (2006). The effect of different bleaching agents on the surface texture of restorative materials. *Operative Dentistry*.

[15] Yu H., Li Q., Wang Y.-N., Cheng H. (2013). Effects of temperature and in-office bleaching agents on surface and subsurface properties of aesthetic restorative materials. *Journal of Dentistry*.

[16] Malkondu Ö., Yurdagüven H., Say E. C., Kazazoğlu E., Soyman M. (2011). Effect of bleaching on microhardness of esthetic restorative materials. *Operative Dentistry*.

[17] Okte Z., Villalta P., García-Godoy F., Lu H., Powers J. M. (2006). Surface hardness of resin composites after staining and bleaching. *Operative Dentistry*.

[18] Basting R. T., Fernandéz Y Fernandéz C., Ambrosano G. M. B., de Campos I. T. (2005). Effects of a 10% carbamide peroxide bleaching agent on roughness and microhardness of packable composite resins. *Journal of Esthetic and Restorative Dentistry*.

[19] Castillo-Ghiotto G., Delgado-Cotrina L., Evangelista-Alva A. (2014). Efectos de la chicha morada y café sobre el esmalte dental bovino blanqueado con peróxido de hidrógeno. *Revista Estomatológica Herediana*.

[20] Ramos-Escudero F., González-Miret M. L., García-Asuero A. (2012). Effect of various extraction systems on the antioxidant activity kinetic and color of extracts from purple corn. *Vitae*.

[21] Kalachandra S., Turner D. T. (1987). Water sorption of polymethacrylate networks: bis-GMA/TEGDM copolymers. *Journal of Biomedical Materials Research*.

[22] Kumar N., Sangi L. (2014). Water sorption, solubility, and resultant change in strength among three resin-based dental composites. *Journal of Investigative and Clinical Dentistry*.

[23] Olms C., Setz J. M. (2013). The repeatability of digital shade measurement—a clinical study. *Clinical Oral Investigations*.

[24] Özat P. B., Tuncel İ., Eroğlu E. (2013). Repeatability and reliability of human eye in visual shade selection. *Journal of Oral Rehabilitation*.

[25] Paul S. J., Peter A., Rodoni L., Pietrobon N. (2004). Conventional visual vs spectrophotometric shade taking for porcelain-fused-to-metal crowns: a clinical comparison. *International Journal of Periodontics and Restorative Dentistry*.

[26] Sikri V. K. (2010). Color: implications in dentistry. *Journal of Conservative Dentistry*.

[27] Ruyter I. E., Nilner K., Möller B. (1987). Color stability of dental composite resin materials for crown and bridge veneers. *Dental Materials*.

[28] Kuhnert N. (2010). Unraveling the structure of the black tea thearubigins. *Archives of Biochemistry and Biophysics*.

[29] Vichi A., Ferrari M., Davidson C. L. (2004). Color and opacity variations in three different resin-based composite products after water aging. *Dental Materials*.

[30] Attin T., Hannig C., Wiegand A., Attin R. (2004). Effect of bleaching on restorative materials and restorations—a systematic review. *Dental Materials*.

